# Enabling patients to cope with psychotropic medication in mental health care: Evaluation and reports of the new inventory MedSupport

**DOI:** 10.1097/MD.0000000000018635

**Published:** 2020-01-03

**Authors:** Karin Drivenes, John-Kåre Vederhus, Vegard Øksendal Haaland, Torleif Ruud, Yina Luk Hauge, Hilde Regevik, Ragnhild Sørum Falk, Lars Tanum

**Affiliations:** a Division of Mental Health and Addiction Services Sørlandet Hospital; b South Eastern Norway Hospital Pharmacy Enterprise, Kristiansand; c Addiction Unit, Sørlandet Hospital; d Clinical Neuroscience Research Group, Department of Psychology, The Faculty of Social Sciences; e Clinic for Health Services Research and Psychiatry, Institute of clinical medicine, University of Oslo; f Department of R&D in Mental health service, Akershus university hospital; g Oslo Centre for Biostatistics and Epidemiology, Research Support Services, Oslo University Hospital, Oslo; h Department of Nursing and Health Promotion, Oslo Metropolitan University, Norway.

**Keywords:** medication, Mental Health, PROMs, support

## Abstract

This cross sectional study examined patients’ perceptions of professional support regarding use of psychotropic medication in a specialist mental health care setting. The aims were to evaluate reliability and validity of the MedSupport inventory, and investigate possible associations between MedSupport scores and patient characteristics.

A cross-sectional study was performed. The patients completed the MedSupport, a newly developed self-reported 6 item questionnaire on a Likert scale ranged 1 to 5 (1 = strongly disagree to 5 = strongly agree), and the Beliefs about Medicines Questionnaire. Diagnosis and treatment information were obtained at the clinical visits and from patient records.

Among the 992 patients recruited, 567 patients (57%) used psychotropic medications, and 514 (91%) of these completed the MedSupport and were included in the study. The MedSupport showed an adequate internal consistency (Cronbach alpha.87; 95% CI.86–89) and a convergent validity toward the available variables. The MedSupport mean score was 3.8 (standard deviation.9, median 3.8). Increasing age and the experience of stronger needs for psychotropic medication were associated with perception of more support to cope with medication, whereas higher concern toward use of psychotropic medication was associated with perception of less support. Patients diagnosed with *behavioral and emotional disorders, onset in childhood and adolescence* perceived more support than patients with *Mood disorders*.

The MedSupport inventory was suitable for assessing the patients’ perceived support from health care service regarding their medication. Awareness of differences in patients’ perceptions might enable the service to provide special measures for patients who perceive insufficient medication support.

## 1 Introduction

Interdisciplinary specialized mental health and addiction services offer treatment for patients with different levels of symptom burdens and impairments in mental functions. During the past few decades, improvements of the health services for patients within this field have focused on increased patient involvements in decisions related to the treatment.^[1–3]^ Clinical work and research within the mental health field have increasingly concerned patient reported outcome measures (PROMs). A PROM is an instrument for patients to report their function and symptoms related to their health and treatment,^[4]^ and concerns the patient views on the outcome, which is an important, independent factor in treatment evaluations.

Modern health care services strive to improve treatment outcomes by engaging patients in treatment plans. Pharmacological interventions have their natural place in mental health services preferably in combination with other treatment modalities.^[5]^ The patients’ existing attitudes to medication have been shown to be important for the treatment. It is also important to what extent the patients receive sufficient information about their medications, and their experience of support in connection with such treatment.^[6,7]^ If the patients have negative attitudes to their medications and simultaneously receives inadequate support in medication issues, they will to a lesser extent be adherent to the treatment. Although medication is helpful to many patients, and has an indisputable place in the treatment of a number of psychiatric illnesses, they might also be contentious and encumbered with disadvantages like side effects, and administration difficulties.^[8]^ More systematic measures to capture patients’ experiences with medication treatments should be used to adjust and correct the individual medication and thereby improve the patients’ compliance. Knowledge about the medication is suggested important, but is often insufficient for patients using antipsychotics.^[9–11]^ A recent study has shown that low adherence to long-term medications is related to negative beliefs about medications and to inadequate information given to patients about their medications.^[12]^ A Norwegian multicenter study was concerned about this as part of their outcome, but could not suggest any satisfactory tool that could measure the patients’ perception of support in connection with the use of medication. As a consequence, the MedSupport inventory was developed to explore how patients perceived support from the mental health service regarding psychotropic medications. We find it important to explore the patients’ perceptions in a structured way, in order to improve their handling of medication and to identify factors relevant for tailored measures.

As the existing Beliefs about medicines questionnaire (BMQ) discloses patients beliefs about medication as an internal attitude,^[13]^ the MedSupport inventory enters the external issue; to which extent the patients perceive support from the service. This study aimed to evaluate the internal consistency of the MedSupport inventory and present data from the first study to use this instrument. Further, we aimed to investigate possible associations between patients’ perceptions of medication support and demographic factors, clinical factors, and patient beliefs about medications. Our hypothesis was that the MedSupport score would be positively correlated with the BMQ-factor regarding needs of medication and negatively correlated with the BMQ-factor about concerns related to use of medications.

## 2 Setting and methods

### 2.1 Context

The health services in Norway are divided into a primary care level and a secondary, interdisciplinary specialist care level, which includes hospital care. Both primary and secondary care services are mainly publicly operated. Sørlandet Hospital provides specialist care for both rural and urban communities, covering a population of 302 000.^[14]^ The Division of Mental Health has 12 different locations that provide ambulatory treatment, day care, in-patient treatment, and out-patient treatment. The division provides general mental health treatments, forensic psychiatry, child and adolescent psychiatry, geriatric psychiatry, and treatments for substance-related disorders for the region. The division holds 280 beds and manages 4150 admissions and 184,000 consultations per year.

### 2.2 Study design and population

A cross-sectional study was carried out at the Division of mental health during the third week in January, 2017. Patients were included consecutively when they had regular treatment courses in the hospital, and were included from visit 2. Day care patients and out-patients were included when they arrived for their regular appointment. Patients who receive ambulatory care were not capable of, or willing to, attend the hospital locations, or ambulatory care gave the best possible utilization of the treatment. They received treatment at home, and were included at their place of recidence during the visit from their therapist. In-patients were included at a scheduled talk after 24 hours of hospitalization. The patients were given information about the study from information posters in the clinics, from the receptionist, and from their therapist. We excluded patients under 16 years of age, patients attending their first consultation, patients that had been admitted for less than 24 hours, and patients that did not speak or read the Norwegian language. Patients were also excluded when inclusion would be harmful to the ongoing treatment or the patient-therapist relationship, or when the patient was considered unable to complete a written questionnaire. Patients were only included once if they were scheduled for more than 1 contact with the service during the study week. The patients were recruited from all parts of the service. They fulfilled the questionnaire during their regular visit, and the questionnaires were collected by the therapist or receptionist directly after the visit. Only patients using psychotropic medications as part of their treatment course and completing the MedSupport were included in this paper.

The patients reported age, gender, use of psychotropic medication, beliefs about medicines (using BMQ-specific which includes the factors needs and concerns regarding medication), and perceived support for medicines (the MedSupport inventory). Information on diagnoses, treatment durations, treatment modalities, and any compulsory treatments were obtained from patient records ().

**Figure 1:**
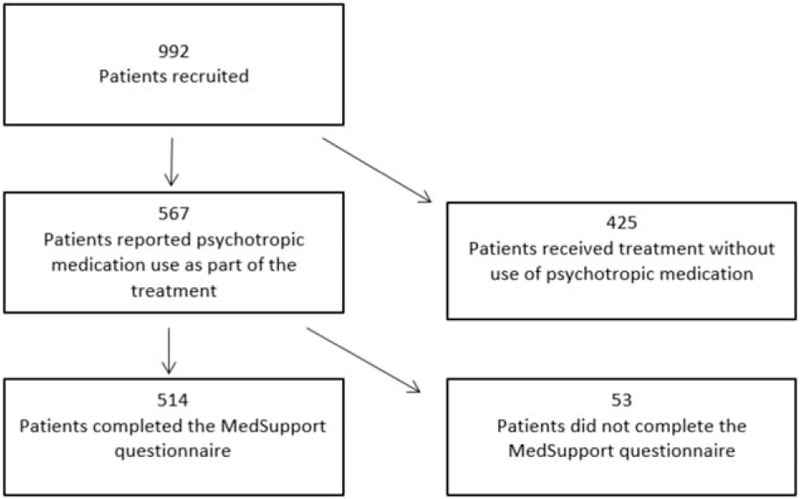
Flow chart of the patients recruited to the study.

The study was approved by the Norwegian Regional Committees for Medical and Health Research Ethics (no 2016/1781) and by the Hospital Board of Research (no: 17/00104). All patients provided written informed consent after receiving oral and written information prior to participation.

### 2.3 Instruments

#### 2.3.1 The MedSupport inventory

**Figure 2:**
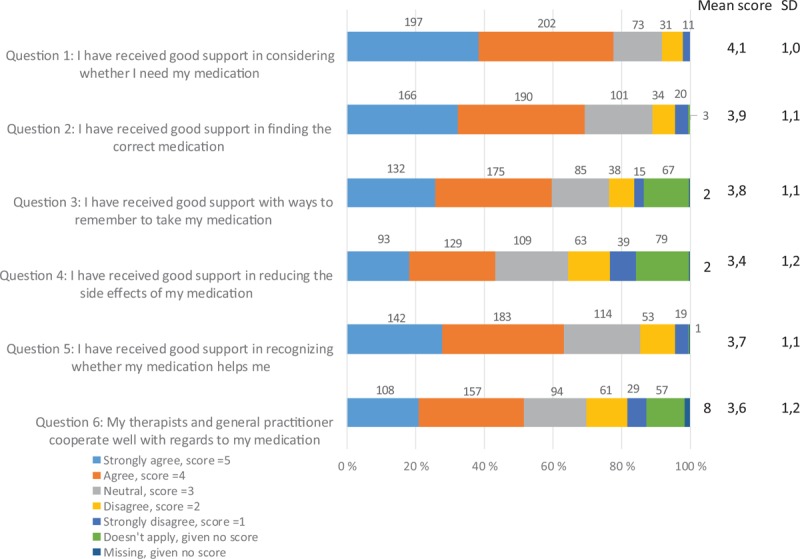
The MedSupport inventory items and the distribution of responses, n = 514.

The MedSupport is a 6-item PROM instrument, constructed by a task force consisting of clinicians and researchers in Norway, to assess whether patients received support in dealing with medications. It was constructed for an ongoing Norwegian multi-center cluster randomized study on the implementation of guidelines and evidence-based treatments of psychoses; however, it has not been previously validated^[15]^ (Fig. 2).

The inventory consists of a 5-point Likert scale, where patients can express how they agree with each statement on a scale with range anchored by 1 (strongly disagree) and 5 (strongly agree), with 3 as a neutral mid-point. An important issue for the task force when constructing the questionnaire was that every item should contain a reasonable alternative for all patients. This premise was fulfilled by providing a “not applicable” box. This is shown as a separate response alternative in Figure 2. A mean score was calculated for the cases where at least 4 questions were answered. A higher mean score indicated that the patients perceived better support for the measured core aspects of using medicines.

The first 5 questions in the MedSupport questionnaire are statements covering core aspects regarding patients’ perceived support with medication, including: experienced need for medication, optimizing psychotropic medication, ways to remember to take medication, reducing the adverse effects, and to recognize improvement of symptoms due to medication. The last question discloses the perceived cooperation between the mental health care service and the general practitioner. For publication purposes the MedSupport was translated into English, and then back-translated by a professional translation company.^[16]^

#### 2.3.2 The Beliefs about Medicines Questionnaire (BMQ)

The BMQ, developed by Horne and coworkers, is an instrument for assessing patients’ beliefs about their medication.^[17]^ It is available in several version for patients and practitioners, and has been translated into Norwegian and validated for use in psychiatric practice.^[1,13,17,18]^ We applied a part of the BMQ inventory, the BMQ-specific, which concerns patients’ present use of medication, and comprises two 5-item factors regarding beliefs about medication prescribed. The factors are the beliefs about the necessity of prescribed medication (BMQ needs), and the beliefs about the danger of dependence and long-term toxicity and the disruptive effects of medication (BMQ concerns).^[17]^ The items are presented as statements and are scored by the patients on a 5-points Likert scale, which ranges from 1 (strongly disagree) to 5 (strongly agree). Calculations of BMQ needs and BMQ concerns were done by summarizing the scores on the 5 corresponding items related to each factor. Higher scores refer to greater beliefs.

### 2.4 Data analyses

The reliability of the MedSupport inventory was tested with Cronbach alpha coefficient to establish an internal consistency. The latent factor structure was identified by exploratory factor analysis with the maximum likelihood technique to test the dimensionality of the MedSupport. Factors were identified by promax oblige rotation. Kaiser eigenvalue-greater-than-one rule was used to determine the number of factors. Pearson correlation was applied to evaluate the concurrent validity of MedSupport by identifying correlations to the scores on BMQ needs and BMQ concerns. We explored discriminate validity by comparing means of MedSupport scores between patients subject to compulsory treatment and voluntarily treated patients.

Descriptive analyses were used to describe the patient population and to examine patients’ perceptions of the support they received for managing their medications. Results were expressed as frequencies, proportions, and means and standard deviations (SD). Associations between perceptions of support expressed by MedSupport scores, and age, gender, diagnosis, any compulsion, treatment durations, and treatment modalities were explored to examine patient characteristics relevant for extent of support regarding medication. This was performed by univariable and multivariable linear regression models.^[19]^ Results were presented as beta coefficients with 95% confidence intervals and *P* values.

Age at inclusion was found to have a linearity relationship with MedSupport, and was presented per 10 years in the regression analyses. Gender (male/female) and compulsory treatment (yes/no) were considered as dichotomized variables. The median treatment duration was used as a cut-off value to distinguish longer- from shorter- term treatments. Treatment was categorized into 4 different modalities: ambulatory care, day care, in-patient care, and out-patient care. Out-patient treatment was set as the reference category as the majority of the included patients were in this group. The main diagnoses were categorized according to the International Statistical Classification of Diseases and Related Health Problems, 10th Revision classification system (ICD-10).^[20]^ The diagnoses were classified as: F10-19 *(Mental and behavioral disorders, due to psychoactive substance use)*, F20-29 *(Schizophrenia, schizotypal, and delusional disorders)*, F30-39 *(Mood disorders)*, F40-48 *(Neurotic, stress-related, and somatoform disorders)*, F60-69 *(Disorders of adult personality and behavio*r), and F90-98 *(Behavioral and emotional disorders, usually with childhood and adolescence onsets)*. The *Mood disorder* group (F30-39) was set as reference diagnostic group in the regression analyses, as it was regarded as a principal diagnosis group among the mental health disorders, and was the most frequent diagnosis group among the included patients. The 2 factors of BMQ-specific, needs and concerns, were analyzed separately in the regression models, as 2 independent continuous variables.

All analyses were performed with Statistical Package for the Social Sciences (SPSS) version 23.^[21]^

## 3 Results

### 3.1 Population characteristics

Among the 992 patients recruited, 567 patients (57%) reported regular use of 1 or more psychotropic medications, and 514 (91%) of these completed the MedSupport questionnaire and were included in the analyses this paper.

The included patients had a mean age of 38.1 years (SD 13.6), and 56% were women. No information about ethnicity was acquired from the patients, but more than 90% were white. The most frequent diagnosis was *Mood disorders* (F30-39) (n = 130 patients; 25%), followed by *Neurotic, stress-related and somatoform disorders* (F40-48) (n = 123; 24%). Only 23 patients (5%) were subjected to compulsory treatment. The mean treatment duration was 6.4 years (SD 7.6, range 0–40 years), with a median of 3.0 years. Out-patient treatment was received by 348 (68%), ambulatory treatment by 57 (11%), day care treatment by 22 (4%), and 82 (16%) were inpatients. The BMQ-specific need score was mean 17.8 (SD 4.5), and the concern score was mean 13.3 (SD 4.3).

### 3.2 MedSupport scores and evaluation of the MedSupport inventory

The mean MedSupport score was 3.8 (SD.9). Question 1 (I have received good support in considering whether I need my medication) had the highest mean score (4.1, SD 1.0); 78% of patients agreed (agree or strongly agree) with the statement. Question 4 (I have received good support in reducing the side effects of my medication) had the lowest mean score (3.4, SD 1.2). Only 43% of patients agreed (agree or strongly agree) with this statement (Fig. 2). The correlation between the items included in the MedSupport were.32 to.73, with the best correlation between the questions 1 (I have received good support in considering whether I need my medication) and 2 (I have received good support in finding the correct medication), and the weakest between questions 1 and 3 (I have received good support with ways to remember to take my medication) ().

**Table 1:**
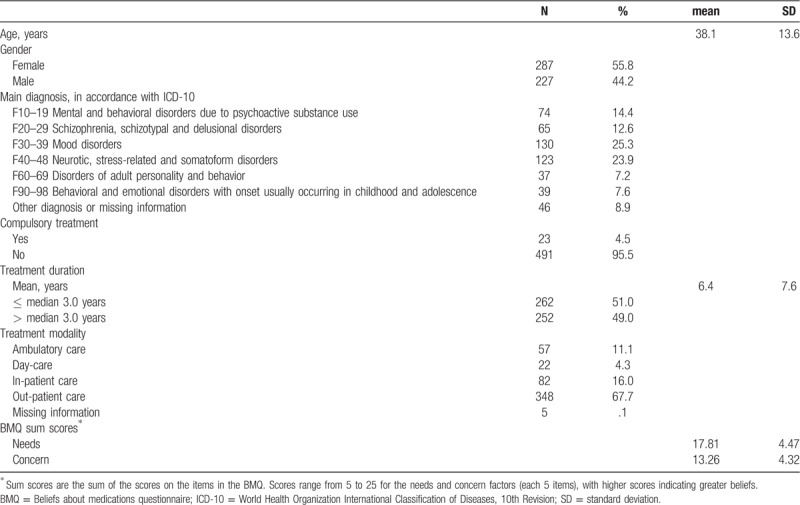
Characteristics of patients included in MedSupport evaluations, n = 514.

The MedSupport questionnaire showed an adequate internal consistency, with a reported Cronbach alpha coefficient of.87 (95% CI.86-.89). From factor analysis the largest Eigenvalue was 3.3 and the second largest was.5, yielding a ratio greater than 3. Thus, the factor structure was in favor of uni-dimensionality. The first factor explained 73% of the variation of the items. As hypothesized, we found a positive correlation between the MedSupport and BMQ needs (.28, *P* < .001) and a negative correlation between the MedSupport and BMQ concerns (−.34, *P* < .001). The mean MedSupport score was.7 lower for compulsory treated patients than for patients receiving voluntary treatment (3.1 and 3.8, respectively, *P* < .001). Data is shown in the univariable regression analysis in Table 2.

**Table 2:**
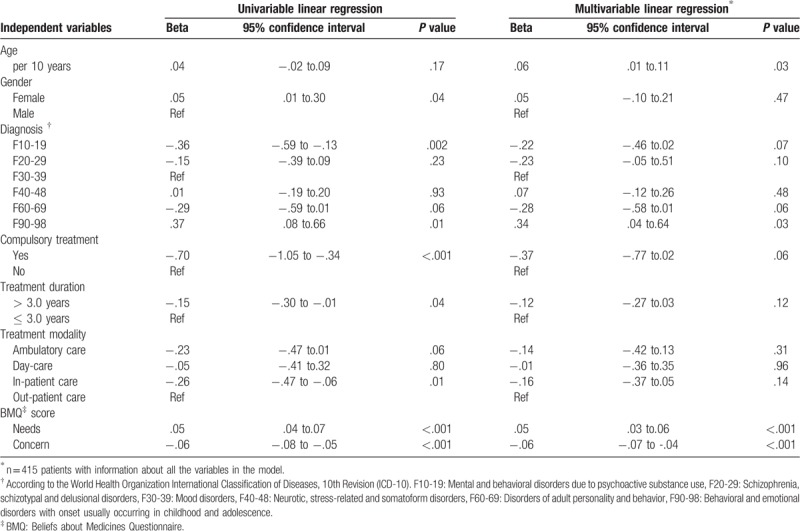
Univariable and multivariable linear regression analyses of MedSupport scores among the included patients, n = 415.

### 3.3 Associations between MedSupport and patient related factors

We found a significant association between the MedSupport and BMQ-specific responses. Reports of a greater belief in the needs of medication were associated with higher MedSupport scores (beta.05, 95% CI.03 to.06, *P* < .001). In contrast, greater concerns about medication use were associated with lower MedSupport scores (beta −.06, 95%CI −.07 to −.04, *P* < .001) (Table 2). Patients at higher age reported more perceived support with their medication than younger patients; an increase of 10 years yielded an average of.06 higher score of MedSupport (*P* = .03).

The patients with *Mood disorders* reported MedSupport scores of mean 3.9 (SD.8, range 1.7–5.0). Explorative analysis showed that this group was the most suitable comparator group for MedSupport scores among the different diagnostic groups. Patients diagnosed with *Behavioral and emotional disorders with onset usually occurring in childhood and adolescence* (F90–98) reported more support than patients with *Mood disorders* (F30–39) (beta.34, 95% CI.04 to.64, *P* = .03). We found no significant association between the other diagnostic groups and the MedSupport scores. There were no significant differences in perceived support between males and females, treatment duration shorter or longer than 3.0 years or between patients subjected to the different treatment modalities (Table 2).

## 4 Discussion

### 4.1 Main findings

We found that the MedSupport inventory had an adequate internal consistency for assessing patients’ perceptions of support from mental health services. It showed a uni-dimensional feature with 1 factor explaining a high proportion of the variance. The convergent validity showed a significant positive correlation between MedSupport scores and BMQ needs although weak, and a significant moderate negative correlation between MedSupport scores and BMQ concerns. The correlation between the questions was significantly moderate to high.

The cross sectional study population showed a diverse pattern with regard to age, diagnosis, compulsion, treatment duration, and treatment modality; reflecting the daily treatment situation at the different clinical units at our hospital. Most of the patients received out-patient treatment, and half of the patients in the study had received treatment for at least 3 years. Good clinical practice implies that such long treatment series include psychoeducation containing information of their medication. Therefore, we expected the included patients to have received information about their medication. However, we could not demonstrate better MedSupport scores among patients with longer treatment durations.

The study sample perceived reasonable support for medication issues, with mean scores on all items above the neutral mid-point on the scale (≥3.4). The lowest score was for the question concerning support in reducing medication adverse effects. Adverse effects are an important reason for discontinuing medication treatments in general,^[22]^ and psychotropic medications might be encumbered with troublesome adverse effects. The lowest score on this question indicates that the service should emphasize focus on reducing the adverse effect burden as much as possible for the individual patients. On the other hand, the question also showed the highest proportion of scores “doesn’t apply”. We suggest an interpretation that this score explains a straightforward experience without problematic side effects from many of the patients. If side effects never was an issue for them, the patients may view the question as irrelevant. Taken together, the variation in the responses to this question may reflect a diverse situation in the clinics, where the majority of patients do not experience adverse effects, but for the patients who do, the service may not take sufficient initiatives and follow-ups, and this group of patients should be a target for closer attention. These patients were identified through the MedSupport, and the inventory may target groups in need for relevant initiatives. Further, the higher proportion of patients reporting “doesn’t apply” to the questions concerning support with remembering to take the medicines and the cooperation between the therapist and the general practitioner, respectively, can also be explained by the patients’ lack of experience of any problem regarding the subjects. Not all patients struggle with remembering their medicines, and a number of patients do not visit their general practitioner on a regular basis.

We accepted questionnaires with at least 4 of the 6 items answered. By doing this we obtained reports also from patients who wished to express their opinion about some of the topics, but not necessarily all topics presented. As more than 90% of the eligible patients answered at least 4 out of the 6 items in the MedSupport, we got a wider basis for exploring their attitudes. The 53 patients who did not complete the MedSupport reported weaker BMQ- specific beliefs about needs and concerns. As they report less need for medication as well as less concerns about negative consequences of medication, we suggest that medication issues are less important to them, and they did not have any motivation to express their opinion with regard to their medication. However, as the patients not completing the MedSupport were more frequently receiving out-patient treatments than the patients who completed the inventory, we suggest the bias was probably partly caused by different inclusion efforts in the different parts of the service.

We found that a greater belief in medication as a necessity correlated positively to the perception of better support. This was in line with our hypothesis: if patients perceive adequate support with their medication and have been educated sufficiently, they will to a greater degree agree on the medication. The finding was supported in a previous study by Horne and Weinman, who found that believing in medication as a necessary part of the treatment promoted adherence, and conversely, concerns about medications hindered adherence.^[13]^ Our findings also indicate that patients’ concern beliefs about medicines reflect the perception of inadequate support with medication issues. This finding is consistent with previous studies, which found patient understanding and acceptance of the treatment (e.g., how to use the medicines) important for medication adherence^[12,23,24]^ and thus, treatment outcome.^[1,5,25]^

Patients diagnosed with F90–98 *Behavioral and emotional disorders with onset usually occurring in childhood and adolescence* reported higher MedSupport scores than the reference population (the patients with F30–39 *Mood disorders*). We propose that there might be a difference in the way the treatment is organized. As the medications frequently used for these conditions are stimulants and categorized as narcotics in the Norwegian prescription system, thorough reviews and tight control are claimed by the authorities. This leads to a more tight follow-up and regular assessments of medication use, which presumably includes all patient medications, and that have a positive effect on the patients’ perceptions of support. Higher patient age was also associated with higher MedSupport scores. This could be an expression of different expectations from older, compared to younger patients to the health care service. Additionally, we would assume that service related factors are involved in this finding as well, i.e. treatment context and practitioner skills.

Mental health care continues to struggle in promoting attitudes and structures that enable patient voices to be heard and acted upon.^[26,27]^ Many of the patients in mental health care are long-term patients, and their experiences with previous treatments were likely to influence experiences with current treatments, which is also mentioned by Mestdagh and Hansen in a qualitative study.^[28]^ Therefore, even though this was emphasized to the patients, the reports were not necessarily related solely to the current treatment, but might include patients’ earlier experiences as well.

Hawkins and co-workers have suggested that PROM evaluations are not limited to the statistical features of the instrument; they include empirical evidence that support its intended use.^[29]^ Due to this, patients reports gain empirical evidence as a supplement to validity exploration of PROMs. Therefore, our patient reports at the MedSupport inventory support the reliability and validity testing to determine application.

Coercion did not affect the perceived support with medication in this study. In the literature patients subjected to compulsory treatment usually differ from voluntarily treated patients in their attitude towards treatment.^[30]^ We do not know the reason behind this unexpected finding, but it could perhaps be coinsidential due to the rather small patient subgroup (n = 23; 4.5%). As we did not perform assessments of adherence or satisfaction in this study, it remains unknown if responses on the MedSupport are directly related to those factors.

### 4.2 Strengths and limitations

A strength of the MedSupport questionnaire is that it is short and easy to apply. Additionally, the MedSupport questionnaire contains global items, i.e. not specific to a specific medicine, diagnosis, or treatment course. Thus, it has a broad applicability for assessing the health care service's ability to support patients who use medicines. As patients differ in education levels, social functioning levels, and illnesses, they require individualized treatment approaches. Another strength is that we included a real world patient population in our study. We will therefore argue the cohort to be considered typical for the population at specialist-level mental health services. Consequently, we suggest our results to be considered valid for the population, despite the limitations.

The psychometric properties examined showed adequate features of the MedSupport inventory. However, it limits the investigation of the MedSupport instrument that this was an explorative study and we did not accomplish a complete validation. We were not able to perform a test-retest to affirm the reliability of the MedSupport. This would have strengthened the reliability of the scale. Additionally, more variables available for comparison to explore construct validity were wanted.

The study had a cross-sectional design from which we cannot draw conclusions about causality, which is a limitation. However, it is likely that the direction of the influence is mainly that support can alter beliefs. Greater support would likely change patient beliefs in a positive direction and conversely, less support would strengthened preexisting negative beliefs (e.g. concern about the medication). Mental health care professionals should preferably conduct explorations of both beliefs and perceived support perspectives, and address revealed issues.

During data collection, we only requested the patients’ main diagnoses. At this demarcation, we missed any secondary diagnoses of potential importance for understanding the patients’ level of functioning, including illicit substance use. The negative influence of comorbid illicit substance use or addiction on patient behavior is well documented for many mental disorders.^[31,32]^ Further, we did not request any assessments of the current severity of the patients’ disorder, which could have provided additional information relevant for the understanding of the concept.

### 4.3 Implications

The MedSupport inventory showed an adequate internal consistency and validity regarding the available variables. It is a brief and easily applicable instrument, which provides knowledge on the perceived support from health care service. We postulate from our findings that by supporting the patients adequately regarding medication issues, their concerns regarding medication can be decreased, and their experience of needs can be increased. Such alterations in beliefs are associated to treatment adherence and treatment outcome. By assessing the patients’ perception of support regarding medication issues, the health care service can tailor their efforts to achieve optimal treatment courses. Further investigation of the inventory would be beneficial to expand the exploration of the associations found in this study.

## Author contributions

KD, VØH and TR composed the MedSupport. KD, JKV, VØH, YLH and HR planned and run the study. YLH collected the data, KD, JKV, YLH and RSF analyzed the data, All authors interpreted the data. KD, JKV, VØH and TL wrote the manuscript. All authors gave contributions, and approved the final manuscript.

Karin Drivenes orcid: 0000-0003-4550-8740.
